# Biomedical Applications of Lanthanide Nanomaterials, for Imaging, Sensing and Therapy

**DOI:** 10.7150/ntno.65530

**Published:** 2022-01-01

**Authors:** Qize Zhang, Stephen O'Brien, Jan Grimm

**Affiliations:** 1 Molecular Pharmacology Program, Memorial Sloan Kettering Cancer Center, New York, NY 10065, USA.; 2 Department of Chemistry and Biochemistry, The City College of New York, 1024 Marshak, 160 Convent Avenue, New York, New York 10031, USA.; 3 Department of Radiology, Memorial Sloan Kettering Cancer Center, New York, NY 10065, USA.; 4 Department of Pharmacology, Weill Cornell Medical College, New York, NY 10021, USA.; 5 Department of Radiology, Weill Cornell Medical College, New York, NY 10021, USA; 6 Ph.D. Program in Chemistry, The Graduate Center of the City University of New York, New York, New York 10016, USA.

**Keywords:** Lanthanide Nanomaterials, Imaging, Sensing, Therapy

## Abstract

The application of nanomaterials made of rare earth elements within biomedical sciences continues to make significant progress. The rare earth elements, also called the lanthanides, play an essential role in modern life through materials and electronics. As we learn more about their utility, function, and underlying physics, we can contemplate extending their applications to biomedicine. This particularly applies to diagnosis and radiation therapy due to their relatively unique features, such as an ultra-wide Stokes shift in the luminescence, variable magnetism and potentially tunable properties, due to the library of lanthanides available and their multivalent oxidation state chemistry. The ability to prepare nanomaterials of relatively smaller sizes has increased the likelihood of use *in vivo*. In this review, we summarize the different emerging applications of nanoparticles with rare earth elements as the host or doped elements for biomedical applications in the past three to four years, especially in the area of imaging and disease diagnosis. Researchers have made progress in utilizing surfactants and polymers to modify the surface of lanthanide nanoparticles to enhance biocompatibility. At the same time, specific antibodies and proteins can also be conjugated to these nanoparticles to increase targeting efficiency for specific tumor models. Finally, in the near-infrared II imaging window, lanthanide nanoparticles have been shown to exhibit extraordinary bright emission, which is an exciting development for image-guided surgery.

## Introduction

This is a short review of the current use of lanthanide nanomaterials for biomedical applications. The word lanthanide is used colloquially over lanthanoid, which is the official IUPAC name. Collectively with Scandium and Yttrium, the term rare earth element (REE) is frequently used in geology, science and medicine; however, their relative natural abundance is not that scarce in most cases. The f-orbitals start with element 57, lanthanum, the first of the lanthanides and namesake of the elementary group. There are so many of these f-block elements (15, from Lanthanum to Lutetium) it is necessary for formatting/aesthetic reasons to place them at the bottom of the periodic table, with the actinides. Their discovery spans the 19^th^ century, while the emergence of their widespread utility spans the latter half of the 20^th^ century until the present day. As we have learned to harness the unique physics and chemistry characterized by their f-orbital behavior, there have been several key applications that have revolutionized industries, including the development of phosphors for fluorescent/LED lighting 1 and the development of rare earth magnets, which now dominate the design of personal headphones, motors for electric/hybrid vehicles, wind turbines, and portable MRI scanners 2-4. Since 1959, when the Dutch-American physicist and Nobel laureate Nicolaas Bloembergen observed nonlinear up-conversion of lanthanides in the Near IR region, optical properties (photonics) research chiefly focused on semiconductors and electronic equipment 5. However, in more recent times, and primarily through adaptation enabled by nanotechnology, significant achievements have been made in basic research of rare earth element chemistry for biomedical applications 6.

In general, molecular-level based imaging is increasingly used for disease diagnosis and guidance of surgery 7-14. Among the most widely used *in vivo* applications are planar and tomographic fluorescence and bioluminescence imaging. However, despite their utility, these techniques are primarily restricted to pre-clinical use. Factors that have prevented translation from the bench to the bedside include depth-penetration considerations, regulatory issues, and toxicity of the lanthanides 15, 16. A recent development in lanthanide element based near IR-II nanoprobes 17 successfully overcame the shortcomings of traditional fluorescence contrast agents by extending the imaging window closer to the near IR-II region (1000-2000 nm). In parallel, nanotechnology researchers dedicated to developing rare earth nanoprobes with improved surface modification, morphology, and size, have enabled increased function for diagnosis or targeting.

In terms of treatment, especially tumor treatment, due to the powerful optical properties of lanthanides, there are remarkable effects when combining them with agents designed for photothermal therapy (PTT) or photodynamic therapy (PDT). Hereafter, we discuss the development of lanthanide element-based nanomaterials in biomedicine, especially for tumor imaging and therapy. Due to several existing excellent reviews on the topic 18-23, we present here exciting recent developments in the past few years. Starting with lanthanide nanoparticle synthesis and surface modification strategies, we summarize what approaches have made them suitable for *in vivo* studies. Next, we evaluate how researchers have utilized the rare earths' unique properties to achieve specific goals in cancer diagnosis, Immunotherapy, chemotherapy, and imaging guide surgery. Lastly, we review applications other than cancer such as, bone imaging, blood vasculature imaging and COVID-19 detection.

## Surface modification of lanthanide-based nanoparticles for *in vivo* studies

**Figure 1:**
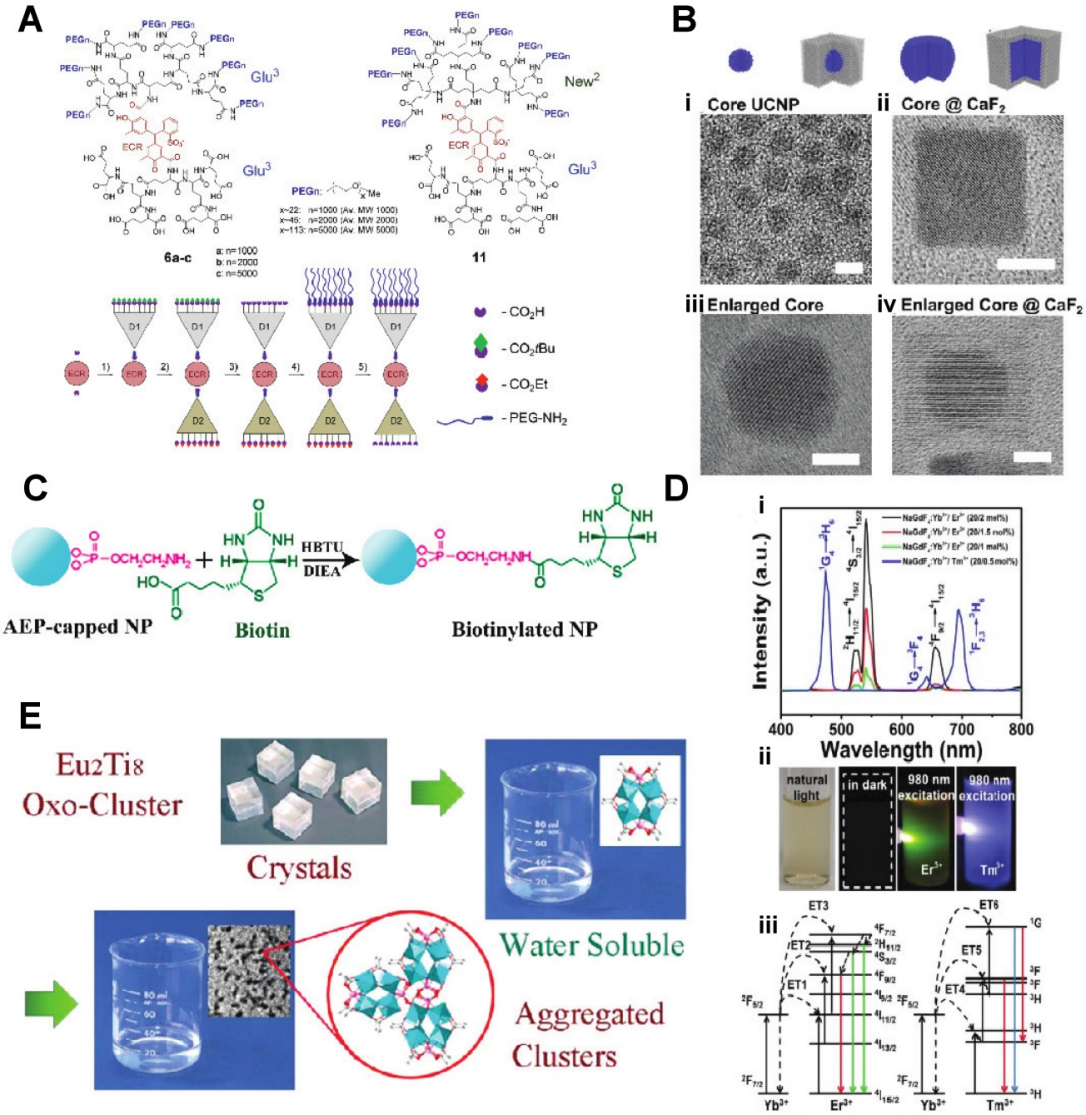
** A)** Janus-type dendritic ligands: Structures and a cartoon of the synthetic sequence illustrating assembly of compound 11 as an example. **B)** Schematics and corresponding transmission electron microscope (TEM) images(i-iv) **C)** The covalent coupling of biotin with NPs can be achieved in DMF solution by use of HBTU and DIEA as coupling reagents. **D)** (i) Upconversion spectra of NaGdF_4_:Yb^3+^/Er^3+^ nanoparticles and NaGdF_4_:Yb^3+^/Tm^3+^ nanoparticles excited with a 980 nm laser. (ii) The digital photographs of colloidal solution of NaGdF_4_:Yb^3+^/Er^3+^(20/2 mol) and NaGdF_4_:Yb^3+^/Tm^3+^(20/0.5 mol) nanoparticles in water under natural light, in dark and excited with a 980 nm laser, respectively. (iii) The energy levels diagrams and corresponding transfer mechanism of Yb^3+^ to Er^3+^ and Yb^3+^ to Tm^3+^. **E)** Water-Soluble Lanthanide-Titanium-Oxo Cluster 10-14.

For pre-clinical nanomedical research, especially in inorganic metal/metal oxide nanoparticles, the highest priority is to enhance biocompatibility. Most biomaterials are considered as foreign matter to the host, especially the inorganic materials, and will induce the rejection reaction. The successful design of an *in vivo* used material must include low toxicity, longer circulation time, and even biodegradable or smaller enough size for extraction from the glomerulus. Making the lanthanide nanomaterial more biocompatible through surface modification became a popular research field for material scientists: Liu et al*.* developed a novel inorganic oxide nanoprobe based on amine-functionalized tetragonal ultrasmall ZrO_2_Ln^3+^ nanoparticles (< 5nm) synthesized through a simple solvothermal technique (Fig. 1C). To substitute the natural hydrophobic ligands on the surface of NPs, amine and phosphate groups were introduced for a ligand exchange reaction. They show the use of this hydrophilic Ln^3+^-based long-lived luminescence as a sensitive, time-resolved fluorescence resonance energy transfer (FRET) nanoprobe to identify avidin with a detection limit as low as 3.0 nM 24. Fischer *et al.* reported synthesizing a series of sub-15 nm alkaline-earth lanthanide fluoride upconversion nanoparticles with different doping combinations (M_1-x_ Ln_x_ F_2+x_, MLnF) (Fig. 1B). Then, CaF_2_ shells, which have earlier been demonstrated to be biocompatible, are employed for passivation to increase the upconversion performance of these nanoparticles by more than five orders of magnitude while also enhancing their biocompatibility 25. Zhijie Wang et al. presented a facile one-pot procedure for producing highly hydrophilic and biocompatible NaGdF_4_:Yb^3+^/Er^3+^ and NaGdF_4_:Yb^3+^/Tm^3+^ nanocrystal by thermal hydrolysis of lanthanide-oleate precursors and sodium fluoride by added ω-dicarboxylic polyethylene glycol (HOOC-PEG-COOH) (Fig. 1D).

Moreover, NaGdF_4_:Yb^3+^/Er^3+^ nanocrystals have been employed effectively as dual-modal imaging contrast agents for optical and MRI imaging *in vivo*^26^. In the recent work by Shane Plunkett et al., the procedure was improved by using Janus-type dendrimers, which have individual carboxylates on the surface of the ligand for conjugation to NaYF_4_-based nanocrystals, doped with Yb^3+^ (20%) and Er^3+^ (20%), neutral polyethylene glycol (PEG) groups for water solubility. They also encapsulate a pH-sensitive dye, Eriochrome, as the center of the dendrimer. These Janus-dendrimers modified NaYF_4_ based nanocrystals were excellent vascular markers, enabling for depth-resolved mapping of specific capillaries in the mice brain achieved a depth of 1000 um under continuous wave (CW) stimulation with a relatively low energies level of no more than 20 mW (Fig. 1A) 27. Another exciting aspect of lanthanide nano oxides is unusual water-solubility, which even could be used to functionalize other materials to render them hydrophilic. In Wen Luo *et al*.'s recent work, they doped the Titanium-Oxo Cluster with Eu(III) , Tb(III), and Yb(III) to form water-soluble lanthanide- titanium- oxo clusters (LnTOCs) which could quickly disperse in aqueous media to form a stable solution with aggregation of the clusters. The photocatalytic behavior, fluorescence sensing, and fluorescent labeling have all been retained and verified through in vitro cell imaging (Fig. 1E) 28.

## Cancer imaging and therapy

**Figure 2:**
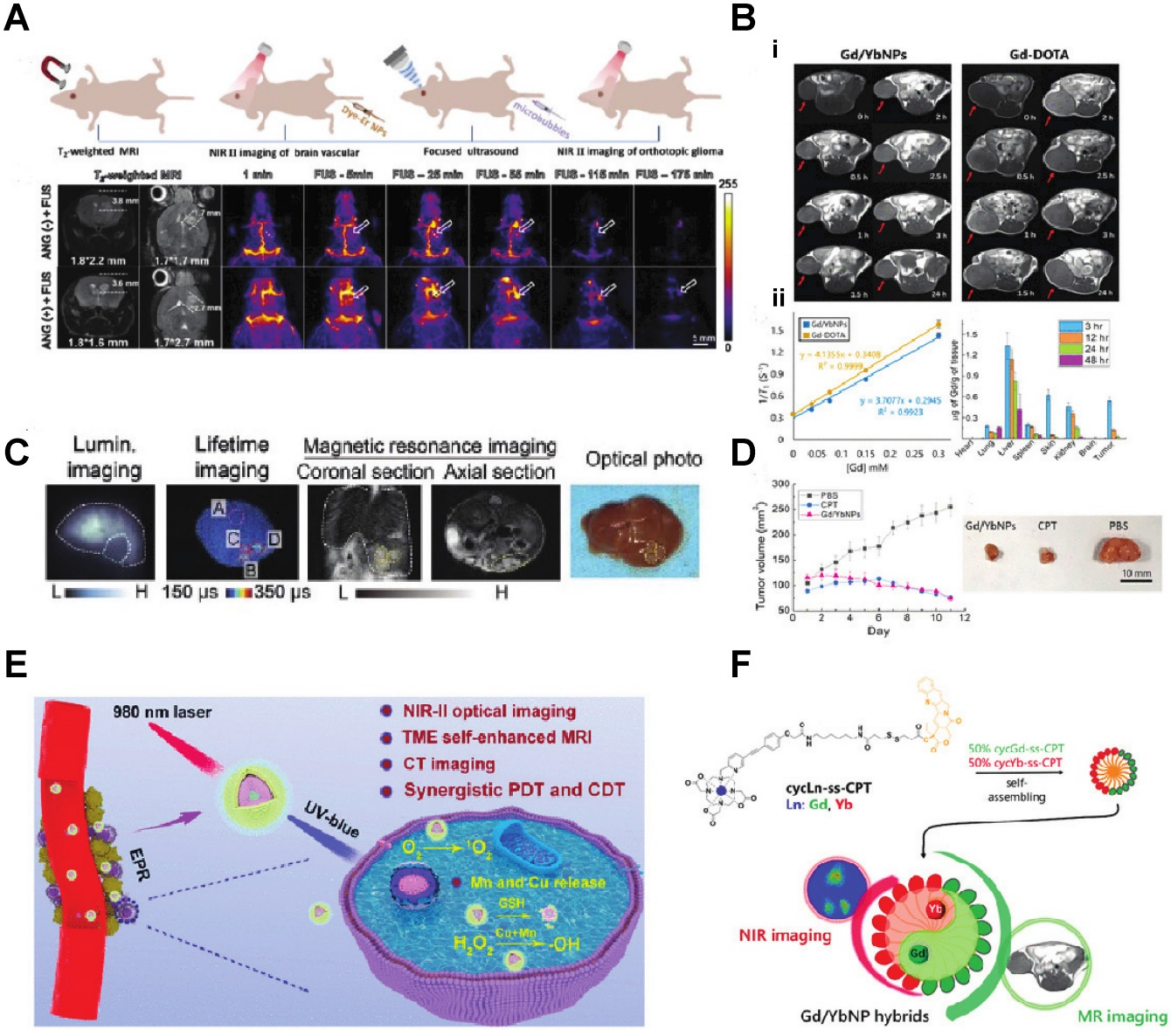
**A)** Schematic illustration of T2-weighted magnetic resonance imaging (MRI), and noninvasive NIR IIb fluorescence imaging of a deep-seated and small-sized orthotopic glioma under the assistance of focused ultrasound (FUS) B, D) (i) *In vivo* T1-weighted MR images of the mice bearing HeLa xenograft from 0 to 24 h after the injection of Gd/YbNPs and Gd DOTA (control). (ii) Plots of 1/T1 vs. [Gd] to determine r1 of Gd-DOTA and Gd/YbNPs hybrid nanoparticles. **C)** Noninvasive intensity-based imaging, lifetime-based imaging of a mouse bearing multiple HCC lesions and optical photo of the dissected liver. **E)** Theranostic mechanism of PEG/ LDNPs@CMSNs for TME and NIR laser co-enabled PDT/CDT and trimodal bioimaging **F)** Lanthanide-Cyclen-Camptothecin Nanocomposites for Cancer Theranostics Guided by Near-Infrared and Magnetic Resonance Imaging 15-19.

Ren *et al.* developed Er-based lanthanide nanoparticles NaErF_4_:2.5 %Ce@NaYbF_4,_ coupled with Dye-brush polymer (Dye-BP) to promote the ^4^I1_3/2_→^4^I1_5/2_ transition, achieved 675-fold enhancement at 1525 nm wavelength near the IR II imaging window. They further conjugated a tumor-targeting angiopep-2 peptide, which could target the glioma in a mice model. Under the guidance of this near IR luminescence probe, they successfully removed the glioma from the mouse brain (Fig. 2A) 29. Peroxynitrite(ONOO^-^) is over-expressed in the tumor-microenvironment. Zhao *et al.* developed a lanthanide-based β-NaYF_4_@NaYF_4_:1%Nd NIR-II nanosensor which attached an ONOO^-^ responsive dye MY-1057. They utilize the specific energy transfer between the ONOO^-^ to the MY1057 dye and finally excite the β-NaYF_4_@NaYF_4_:1%Nd nanosensor in the NIR-II region. With the NIR light from the nanosensor, tumor lesions (Hepatocellular Carcinoma) could be distinguished from normal tissue (Fig. 2C) 30. PDT and CDT (chemodynamic therapy) is usually limited by glutathione and hypoxia in the tumor. Jiating Xu et al. developed a copper/manganese silicate nanosphere (CMSN)-covered by Yb^3+^/Er^3+^/Tm^3+^shell nanoparticles which not only provide upconversion (UC) and downconversion (DC) emissions for NIR (1000 nm - 1700 nm wavelength, the significance of which is explained later in this section) imaging but also contribute to enhancing the potential for PDT through promoting the O_2_ to singlet ^1^O_2_ by the NIR light emitting from the lanthanide element (Fig. 2E) 31.

**Figure 3:**
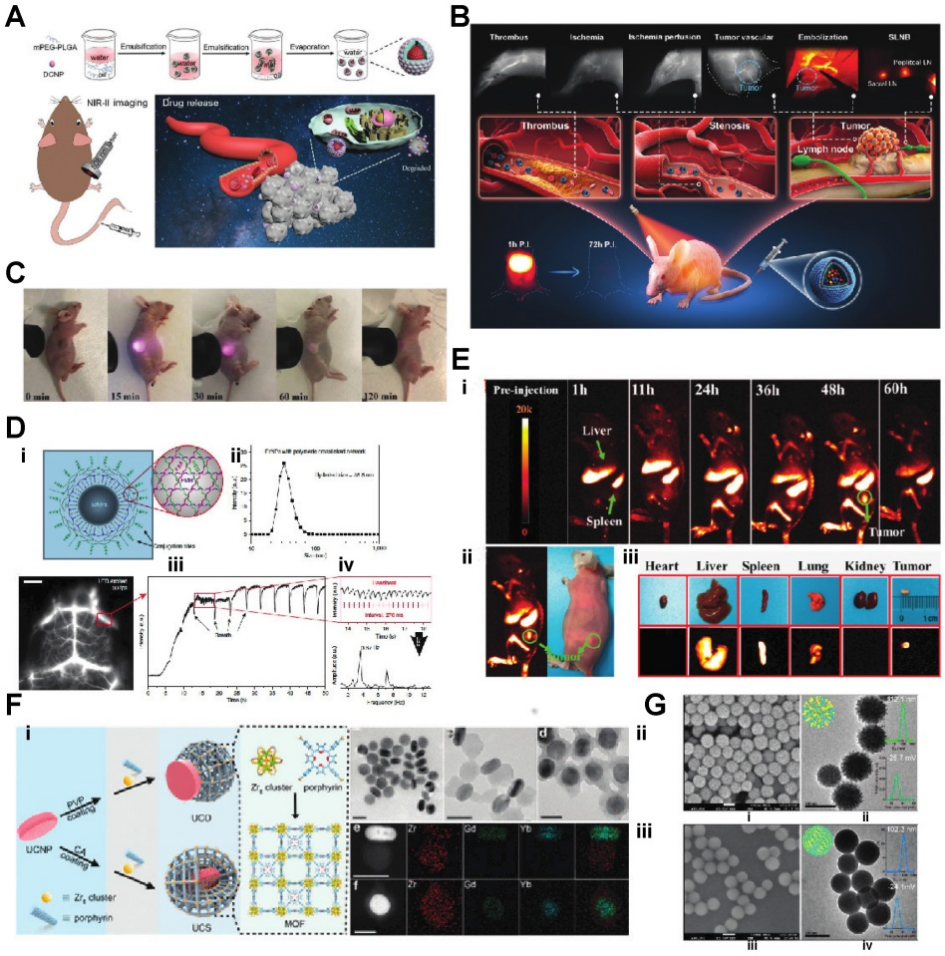
**A)** Schematic illustration of the bioapplication of DMDNs(DOX@mPEG-PLGA@DCNP nanoparticles). **B)** Schematic illustration of excretable lanthanide nanoparticles for multifunctional biomedical imaging and surgical navigation in the second near-infrared window. **C)** Treated group: tumor-bearing mice injected with UCNP@P- RGD-NGR. Digital photos were taken at 0, 15, 30, 60, 120 min after injection under 980 nm excitation.**D**) i), Schematic illustration of the hydrophilic ErNPs with cross-linking polymeric layers and amine groups on the surface as conjugation sites. ii), DLS spectra of hydrophilic ErNPs with polymeric cross-linked network and NIR-IIb cerebral vascular image (left) by intravenous injection of 200 μl ErNPs (40 mg ml-1) and excitation by a 970 nm LED (30 fps). The luminescence intensity of an inferior cerebral vein was plotted as a function of time (iii), showing the cardiac cycles (iv) with a heartbeat frequency of 3.67 Hz by fast Fourier transformation (FFT, lower right). Scale bar, 5 mm **E)** i) Digital photograph of tumor-bearing mouse and *in vivo* NIR-IIb fluorescent imaging (the green circle indicated the tumor site). ii, iii) Digital photographs of the isolated organs/ tumor and the corresponding ex vivo NIR-IIb imaging, respectively. **F)** i) Schematic illustration of the synthesis of UCDs and UCSs through the conditional surface engineering of UCNPs. The structure of porphyrinic MOFs is illustrated in the right column. ii)TEM images from left to right is UCNPs, UCDs, and UCSs. Scale bar, 50 nm. iii) Elemental mapping images of a single (left to right) UCD and (f) UCS. Scale bar, 50 nm. G) (i-iv) Morphology of NdIII-IP virus-like nanodrugs and IP nanospheres observed by SEM, TEM 21-29.

Since some of the lanthanides are also paramagnetic, they can be used as MRI contrast agents. Zhang *et al.* synthesized Lanthanide-Cyclen-Camptothecin nanocomposites encapsulating Gd(III) and Yb(III) complexes and the anticancer drug camptothecin into a micelle, offering real-time NIR / MR dual model imaging and chemotherapy (Fig. 2B, D) 32. Lanthanide nanoparticles could be used as a platform incorporated with other nanomedicines for theranostic approaches. Xu *et al.* integrated CuS nanoparticles (PTT agents) and g-C_3_N_4_ QDs (PDT agents) on silica-coated NaGdF_4_:Yb,Tm@NaGdF_4_:Nd,Yb UCNPs. When they applied an 808 nm laser to this nanocomplex, the lanthanide UCNP would shift the light to UV/vis region and simultaneously active the PPT and PDT agent to achieve the effect of combination therapy 33. Similarly, another group of researchers utilized the same material and 808 nm laser for image-guided tumor resection surgery by doping Ce^3+.^ They achieved an 11-fold enhancement of the NIR-II emission above 1500nm and successfully removed tumors in a colorectal cancer mice model 34. Doping lanthanide elements into polymer nanoparticles is another strategy for cancer imaging or therapy since most organic/polymer nanoparticles can degrade over specific pH environments. Miao Feng et al. introduced hydrophobic NaGdF_4_:2%Nd@NaLuF_4_ nanocrystals ultrasmall nanoparticle and doxorubicin into a 300 nm pH-sensitive degradable mPEG-PLGA nanomicelles for imaging and chemotherapy application (Fig. 3A) 35. While these approaches all used nontargeted agents, lanthanide nanoparticles were also targeted to a specific cancer model for imaging when coupled with targeted peptides. Jingxue Cao et al. designed a αvβ3/αvβ5 and aminopeptidase-N-receptor targeting peptides conjugated to NaYF_4_:Yb nanoparticle as NIR-II nanoprobes for *in vivo* imaging of lung cancer (Fig. 3C) 36. To maximally lower the toxicity of the inorganic lanthanide nanoparticle *in vivo*, an excretable lanthanide nanoprobe for imaging and surgical guidance in the NIR-II window has been developed by Daifen Li and his colleagues (Fig. 3B). They encapsulate ultra-small hydrophobic NaYF_4_:Nd 7% into bio-degradable liposomes. This strategy can quickly clear their particle via the kidneys after intravenous injection with a half-life of 23.0 h for the liver and 14.9 h for the spleen 37. Metal-organic frameworks (MOFs) are posited as excellent photosensitizers for photodynamic therapy (PDT) in cancer therapy. However, the efficacy of the MOFs PDT has been limited by the tissue penetration of the visible excitation light (Fig. 3F).

When coupled with upconversion lanthanide nanoparticles, a more penetrating NIR light source outside the body can be used. The NIR light can be transferred to visible light via upconversion lanthanide nanoparticles inside the body, which could further excite the MOFs to generate singlet oxygen and significantly increase PDT efficacy 38. As an alternative to upconversion, one can also manipulate lanthanide nanoparticles for a down-converting application. Downconversion means shifting the UV/visible (200-400nm) or near IR (800-1000nm) to longer red light (600-700nm) or far IR region (beyond 1500 nm), respectively. Hongjie Dai's group at Stanford designed a biocompatible cubic-phase (α-phase) erbium-based lanthanide nanoparticle attached with anti-PD-L1 antibodies that exhibits bright downconversion emission at ~1,600 nm for dynamic imaging of cancer immunotherapy in mice (Fig. 3D) 39. Another group successfully converted a 980 nm laser emission to 1500 nm (NIR-II region) via poly(acrylic acid) (PAA)-modified NaLnF_4_:40Gd/20Yb/2Er nanorods for cancer metastasis and vessel imaging (Fig. 3E) 40. Downconverting technology could even be applied to nuclear imaging, especially Cerenkov imaging. A europium oxide nanoparticle was utilized to shift blue Cerenkov radiation to the more penetrative red-light region, which significantly enhanced the Cerenkov light for lymph node and tumor imaging 41, 42.

Researchers have also designed rare-earth nanoparticles with different morphologies, resulting in unexpected and interesting effects under certain circumstances. Yang Li et al. created a virus-like nanoconjugate that included a NIR-II fluorescence agent (IR825), a chemotherapy drug (pemetrexed, PEM), and a lanthanide element (Nd(III))(Fig. 3G). The spike-like poly(ethylene glycol) shell protected the nanoparticle from immune clearance and enhanced the *in vivo* circulation time 43. Xu et al. produced two-dimensional ultrathin lanthanide oxyiodide nanosheets for anticancer drug delivery. Those nanosheets exhibit remarkable drug loading capacity (300 wt.%) compared to other nanomaterial morphologies 44. The unique paramagnetic property of gadolinium makes it an excellent MRI contrast agent among rare earth elements. Traditionally, Gd is usually chelated to small molecules. However, material scientists could also make a nanoparticle that uses Gd as the main or doped component. With the benefit of nanotechnology, the Gd nanoparticle could reach the targeted area, relying on the EPR effect, or the particle could be encapsulated with other functional elements to achieve different imaging modalities. Ye Kuang and colleagues doped Gd into HfO_2_ to create a Gd_2_Hf_2_O_7_ nanoparticle that utilizes the Gd as an MRI contrast agent and Hf as a radiosensitizer. They surface modified the nanoparticle with RGD peptides and loaded the particle with cisplatin to a create a potential MRI-guided combined chemo-/photothermal-/radiotherapy nanoplatform for drug-resistant tumors 45. McLeod *et al*. incorporated a Gd(III) complex into a Zr-based MOF (Metal-organic framework) through solvent-assisted ligand incorporation (SALI) and tested the combined MOFs as a potential MRI contrast agent 46. By combining Gd and barium titanate, researchers Peng Wang et al. created a dual MRI and X-ray imaging agent 47. Meanwhile, Yao Cai *et al*. labeled human heavy chain ferritin with Gd for enhanced MRI tumor imaging applications 48. The most abundant lanthanide element, Cerium, is another potential candidate for biomedical applications such as sensors or imaging platforms. With the surface oxygen vacancies, CeO_2_ has been utilized as a contract agent or carrier for another rare earth element for imaging purposes. CeO_2_ also presents native fluorescence due to the presence of Ce^3+^ 49. Meanwhile, CeO_2_ can also be used as a convenient host lattice with which to combine with another lanthanide element to achieve more imaging modalities. Anne et al. doped ytterbium and gadolinium into the CeO_2_ nano cubes for MRI and Near IR imaging on cancer cells 50. Chulun Shao et al. utilize the abundant oxygen vacancies on the CeO_2_ surface and integrate the Gd for DCE-PWI (dynamic contrast-enhanced perfusion-weighted imaging) MRI 51. Since lanthanide elements are in the d-block of the periodic table, they are also considered as high atomic number elements, which usually provide a good contrast for X-ray imaging. Zuwu Wei et al. developed a hollow CeO_2_-ZrO_2_ hybrid nanoplatform labeled with Gd for MRI/CT imaging and drug (Dox) delivery for heterotopic, subcutaneous human liver cancers 52.

## Radiotherapy of ^177^ Lutetium

Lutetium is a unique member of the lanthanide family since one of its isotopes, Lutetium 177, intrinsically has electron and high energy gamma emission, making it a good candidate for targeted radiotherapy. ^177^ Lu has been used as a radiotherapy agent for a long time in the literature 53, and more recently has been chelated to small molecule peptides or prostate-specific membrane antigen (PSMA) inhibitors for targeted radiotherapy of neuroendocrine tumors and prostate cancers. Here, we touch on a subsection of the unique applications of ^177^Lu combined nanotechnology in recent years. Rodrigo et al. recently synthesized an Eu-doped Mesoporous SiO_2_ labeled with ^177^ Lu for HT-29 colorectal cancer imaging and radiotherapy. The author cleverly uses the beta emission from the ^177^Lu for a therapy study, and the gamma emission for SPECT imaging 54. Another group at the University of Wisconsin (Yu *et al*.) developed a facile approach to assembling ^177^Lu-Porphyrin-PEG-Nanocomplexes which combined fluorescence imaging, photodynamic therapy with positron emission tomography imaging, internal radiotherapy and specifically targeted the tumor cell mitochondria 55. Metal nanoparticles could also be labeled with ^177^Lu for therapy study. Pei Pei et al. chelated gold nanoclusters with ^177^Lu and ^99m^Tc, and could strengthen the radioisotope therapy efficiency and induce anticancer immunity by activating dendritic cells 56.

## Biosensing and other biomedical applications of lanthanide nanomaterials

**Figure 4:**
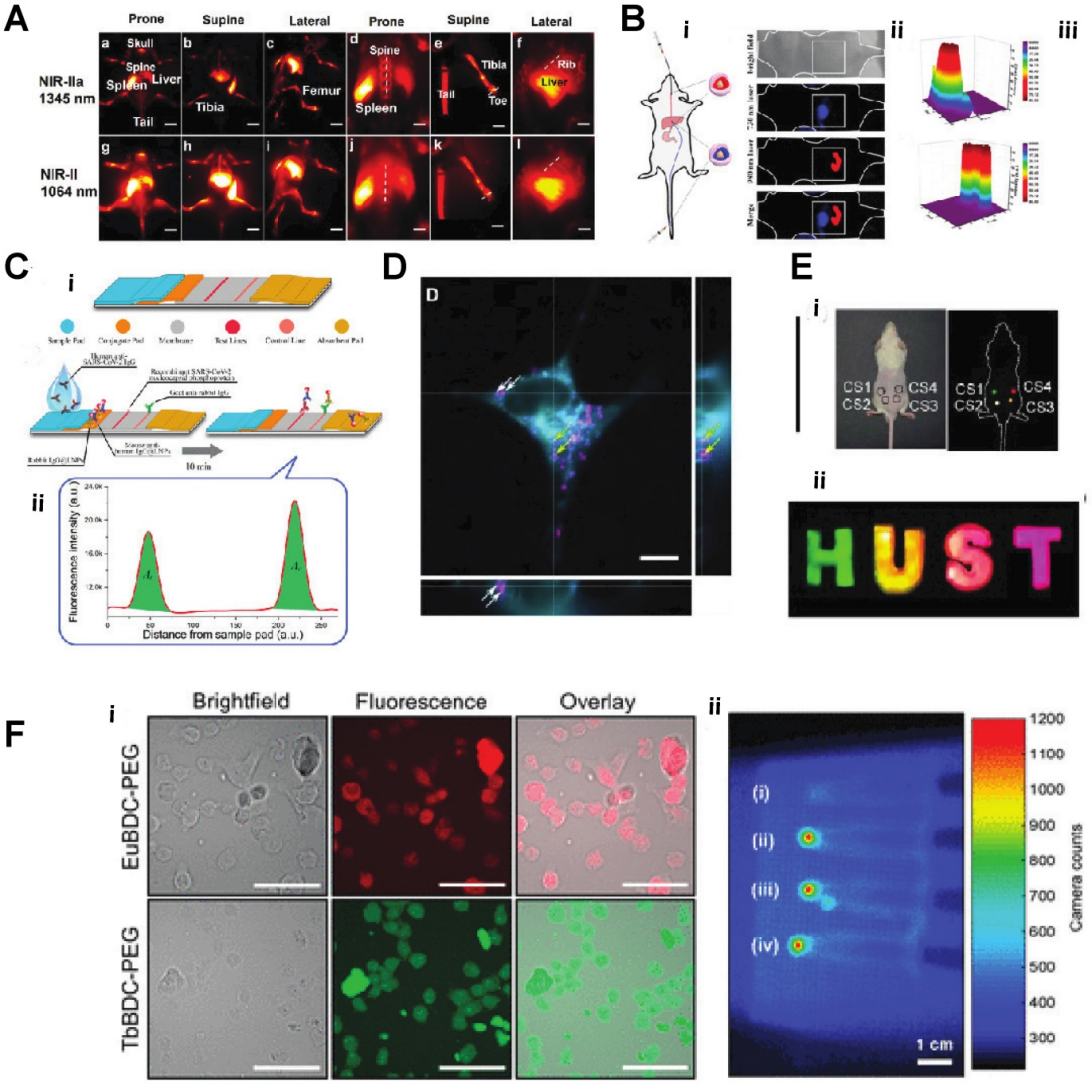
**A)** *In vivo* NIR-II imaging of C57BL/6 mice (n = 3) bone in the NIR-IIa 1345 nm and NIR-II 1064 nm windows. *In vivo* fluorescence of the whole body in (a) prone, (b) supine, and (c) lateral. **B)** Schematic diagram of a nude mouse by intragastric administration and intravenous injection with cssEr-PEG and cssNd-PEG aqueous dispersions, respectively.** C)** Design and fabrication of the developed assay. (i) Lateral flow test strip. (ii) Assay. **D)** Projections of a z-stack of images of a HeLa cell incubated with ECP-NPs (PMMA-COOH 10% at 40% [Eu(tta)3phen]). ECP-NPs are shown in magenta and the cell membranes in turquoise (costained with DiD). **E)** Photograph of papery patterns under 1540 nm excitation (power density: 10 W/cm2). (i) *In vivo* multiplexing upconversion imaging with aqueous CS-CS4 nanoparticles under 1540 nm excitation. Bright-field (left) and dark-field (right) (power density: 0.5 W/cm2), ii. **F)** Luminescence cell imaging of Ln-nMOFs. (above) Confocal images of the CT26 cells incubated with Eu-BDC-PEG and Tb-BDC-PEG nMOFs for 3h. green and red fluorescence represent the emission from Tb^3+^ and Eu^3+^ the PEGylated nMOFs, respectively. Scale bars correspond to 50 μm. (below) In vitro RLI of CT26 cell pellets under X-ray excitation of (i) blank cells and cells incubated with (ii) EuBDC-PEG, (iii)TbBDC-PEG, and (iv) EuxTbxBDC-PEG nMOFs for 24 h 30-36.

**Figure 5:**
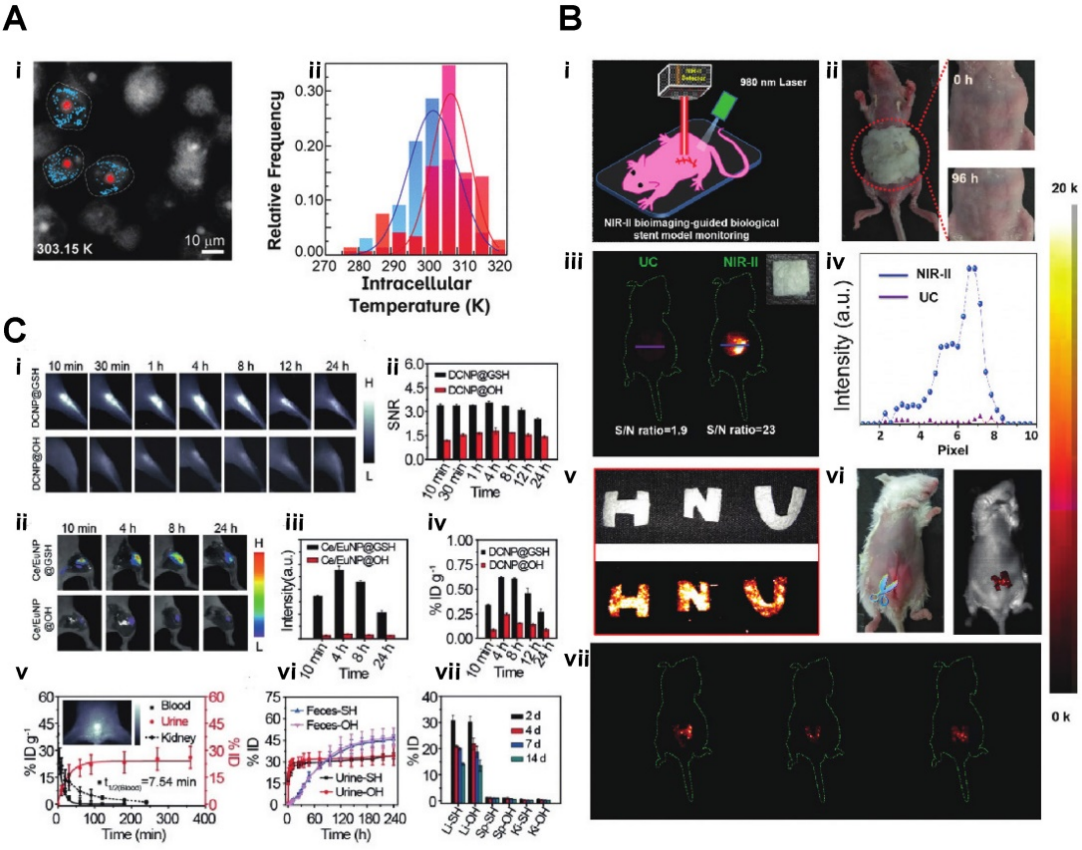
** A)** Microscopy images (recorded in the Eu^3+^ channel) of the MDA-MB-468 cells incubated with DNPD-based polymeric micelles. i)The blue and red points mark, respectively, the locations used for temperature determination of the darker and brighter regions. ii)Temperature histograms obtained from the selected points indicated in panels **B)** i) A schematic illustration of the *in vivo* NIR-II imaging of the implanted biological stent model made from the hybrid silks. ii) Allergy test of a mouse by directly exposing the abdomen skin to the hybrid silks for 96 h. iii. *In vivo* UC/NIR-II imaging of the subcutaneously implanted stent model with a square shape made from the hybrid silk under 980 nm laser excitation. iv)The corresponding fluorescence intensity. v) A digital photograph and in vitro phantom NIR-II imaging of other phantom biological stents with ''H'', ''N'', and ''U'' shapes. vi and vii. *In vivo* NIR-II imaging after subcutaneously implanting the models in the mouse abdomen under the excitation of a 980 nm laser C**,)** i) NIR-II fluorescence bioimaging of the acute local epidermal inflammation with various nanoprobes. ii) SNR as a function of time in the inflamed area. iii) *In vivo* I-PEM bioimaging. iv) I-PEM signal intensity as a function of time in the inflamed area with various nanoprobes. v) ICP-MS results of the retention of various nanoprobes in the inflamed area. vi) Pharmacokinetics of DCNP@GSH in blood and urine excretion after iv) Injection. ICP-MS results of kidney biodistribution as a function of time. Insert shows NIR-II bioimaging of the bladder. vii) Excretion of DCNP@GSH and DCNP@OH in urine and feces as a function of time. h) Biodistribution of DCNP@GSH and DCNP@OH nanoprobes in the liver (Li), spleen (Sp), and kidney (Ki) after two weeks 37-39.

Based upon excellent optical features, such as low background fluorescence, and high sensitivity, lanthanide nanomaterials can be applied to various biosensing areas, such as pH detection, essential physiological ions, ROS detection, temperature monitoring or even COVID-19 testing**.** A current, pandemic related application of lanthanide nanoparticles is the utilization of self-assembled LNPs (lanthanide-doped polystyrene nanoparticles) as a fluorescent sensor to detect anti-SARS-CoV‑2 IgG (Fig. 4C) 57. Inflammation imaging has become, next to cancer imaging, an essential diagnostic tool for disease detection. Zhao et al. developed an ultra-small glutathione (GSH)-modified lanthanide downconversion nanoparticle system that could precisely target and detect ROS in the inflammation through a cross-linking strategy (Fig. 5C). Light and heat have always been a pair of inseparable factors. The intracellular thermal energy could affect the energy transfer of the outer shell electron of the lanthanide element. The outer shell electron configuration usually determines the light emission intensity of the rare earth element. Piñol *et al.* employed this theory to develop innovative Ln^3+^ doped polymeric micelles, which could achieve real-time 2D mapping of the intracellular temperature of breast metastatic adenocarcinoma cells (Fig. 5B) 59.

Similarly, the Mott-Seitz model could describe the light and temperature relationship as equation (1),

Mott-Seitz Model 
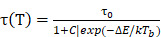
 (1)

where T is the thermodynamic temperature in K, T_0_ is radiative lifetime, E the activation energy of the thermal-quenching effect, and k_b_ is the Boltzmann constant. By applying this theory, Yuen Pan et al. created Eu^2+^/Eu^3+^ co-doped Sc_2_O_3_ nanoparticles, which precisely monitor the temperature changing through the light emission intensity. The unique part of this study was using Eu^2+^ as the primary lattice host rather than Eu^3+^. Due to the lack of complete 5s and 5p orbitals protection, the Eu^2+ 4^f_7_ electron configuration exhibits more sensitivity to the exterior environment, such as temperature 60. Jianan Liu et al. integrate the upconversion nanoparticle with a commercial K^+^ indication into a hollow mesopores silica nanoparticle to create a potassium ion sensor for *in vivo* potassium imaging. By irradiating the NIR light to the upconversion nanoparticle, the NIR light can be transferred to UV light, further activating the K indicator to detect the intracellular K^+^ level 61. Chantal Galaup et al. evaluated a possibility of luminescent lanthanide material that could detect ROS in the context of Alzheimer's disease 62. Cyrille Charpentier et al. designed a particular ligand that could boost the brightness of the terbium nanoparticles and link the nanoparticle to different biological molecules to form specific biosensors to detect biotinylated dyes or even epidermal growth factor receptors (EGFR) 63. For more biomedical applications, Shuqing He *et al*. found a unique property of DSPE-mPEG encapsulated rare-earth-doped nanoparticles, which surprisingly exhibited a high affinity to the bone. They used this particle for dedicated imaging of the bone and lymph nodes (Fig. 4A). The mechanism of this bone affinity is mainly caused by the hydroxyapatite mineral binding ability and white blood cell uptake 64. Rather than laser excitation, lanthanide nanoparticles could also be excited by ionization radiation, like Cerenkov radiation, X-rays etc. 41, 42. Neufeld *et al*., using X-ray as a radiation source, successfully excited nanoscale Ln-MOFs (Ln = Eu or Tb) that emit at 400-700 for radioluminescent cell imaging (Fig. 4F) 65. Another benefit of lanthanide nanoparticles is the long luminescent half-life, eliminating the influence of autofluorescence from body tissue. Andreas Reisch's group developed poly(methyl methacrylate)-based particles doped with a high percentage of europium complexes. By utilizing these rare earth nano complexes, they achieved background-free single-molecule imaging in live cells (Fig. 4D) 66. By adjusting the doping percentage of different lanthanide element components, the emission of the lanthanide nanoparticle could easily be turned into the desired range. With this feature, the same wavelength excitation source could be used to achieve different emission wavelengths for multichannel or multiplexed imaging (Fig. 4B, E) 67, 68.

In an innovative and unusual exploration, Zhiming Deng *et al*. fed silkworms with NaYF4:Gd^3+^/Yb^3+^/Er^3+^@SiO_2_ nanocrystals to produce a NIR-II fluorescent silk fibroin-based material (Fig. 5B). They further bioengineered the upconversion silk by attaching it to an implantable stent for mice to verify the potential for *in vivo* applications 69.

**Toxicity** remains a primary challenge for *in vivo* inorganic nanomedicine, and the lanthanide nanoparticles are no exception 70. Gadolinium chelates are frequently used as MRI contrast agents and recently have been identified as causing renal impairment through slowly developing nephrogenic systemic fibrosis (NSF) in humans that had received certain gadolinium-based MR contrast agents 71. The FDA also issued a safety warning for gadolinium-based contrast agents chelated by linear chelators such as DTPA after observations that Gd could be retained inside the body, even in the brain, for months or even years after the MRI scan. Other studies have shown there is nephrotoxicity of gadolinium-based contrast agents 72. Meanwhile, other lanthanide ions, like Tb^3+^ Pm^3+^ or Eu^3+^ could bind to the Ca^2+^ binding sites of the intestinal membrane, affecting ion channel transportation, leading to toxicity. Sc^3+^, Y^3+^, and La^+^ have been found to bind to globulin and DNA, which could cause damage 73. Encapsulating lanthanides into rapidly excretable nanoparticles and/or coating with a biocompatible polymer which decreases the time of the lanthanide inside the body, can minimize toxicity. Dana M. et al. tested their NaGdF_4_:Tm^3+^ Yb^3+^ nanoparticles on a variety of cancer cell lines and C.elegans. detected no toxicity over a wide concentration range 74. For the same kind of particle, Huaiyong Xing et al. showed after seven days post-injection to the mice, the NaGdF_4_:Tm^3+^ ultra-small nanoparticle could be excreted into feces and urine 75. Jin Yan et al. developed a safe peptide-lanthanide nanocluster for cancer drug delivery. They functionalized 5 nm lanthanide cores with two types of targeting peptide, which increased the tumor-targeting efficacy and decreased the toxicity of the nano cluster 76. Based on the issue of toxicity and the development of strategies to eliminate concerns with introducing lanthanides into the body, the design of lanthanide nanomaterials for biomedical applications continues to focus on developing ultra-small and clearable nanoparticle approaches, while still seeking to take advantage of the available optical properties to enhance sensitivity for disease diagnosis and future innovation in treatment of disease 35, 37, 41.

## Conclusion and Prospects

Biomedical imaging is a multidisciplinary research field involving physics, chemistry, molecular biology, photonics and materials science. In this rapidly developing research field, new materials, new technologies, and new applications arise quickly. Due to the long time for clinical translation, it is essential that biomedical researchers become aware of new materials opportunities that enable improved sensitivity in diagnostic medicine, and increased efficacy/reduced toxicity in therapeutics. This review provided a short summary of the applications of lanthanide nanomaterials published over the past few years, including cell imaging, living tumor diagnosis, lymphatic and vascular imaging, and imaging guide surgery. Given the extensive literature on this global research topic, it is difficult to cover all aspects of rare earth nanomaterial science and biomedical applications. Lanthanide luminescent nanomaterials have made outstanding contributions to the field of biomedicine in the past few decades, mainly due to the apparent advantages of rare earth luminescent materials, such as stable photochemical properties, negligible photobleaching, and high fluorescence to noise ratio. The excitation light wavelength is in the ideal biological window (NIR region), which has better penetration, no damage to the normal tissue and high sensitivity. Because of the unique properties of rare earth luminescent materials, they could provide advantages that other probes cannot achieve and a valuable technical method to biologists and physicians. Optical imaging of rare earth materials is essential for various technologies required for modern bioanalysis and bioimaging. It has allowed for the creation of unusual nanosensors based on the unique properties of the lanthanides. Overall, lanthanide-based agents have shown remarkable application prospects in fluorescence imaging, medical diagnosis, and treatment.

## References

[1] Spiess E, Bruning A, Gack S, Ulbricht B, Spring H, Trefz G (1994). Cathepsin B activity in human lung tumor cell lines: ultrastructural localization, pH sensitivity, and inhibitor status at the cellular level. J Histochem Cytochem.

[2] Ronda CR, Jüstel T, Nikol H (1998). Rare earth phosphors: fundamentals and applications. Journal of Alloys and Compounds.

[3] Coey JMD (2020). Perspective and Prospects for Rare Earth Permanent Magnets. Engineering.

[4] K.H (2001). Jürgen Buschow RWC, Merton C. Flemings, Bernhard Ilschner, Edward J. Kramer, Subhash Mahajan, Patrick Veyssière. Encyclopedia of Materials: Science and Technology, Elsevier,: Elsevier Wordmark.

[5] Bloembergen N (1959). Solid State Infrared Quantum Counters. Physical Review Letters.

[6] Bünzli J-CG (2010). Lanthanide Luminescence for Biomedical Analyses and Imaging. Chemical Reviews.

[7] Chi C, Du Y, Ye J, Kou D, Qiu J, Wang J (2014). Intraoperative imaging-guided cancer surgery: from current fluorescence molecular imaging methods to future multi-modality imaging technology. Theranostics.

[8] Massoud TF, Gambhir SS (2003). Molecular imaging in living subjects: seeing fundamental biological processes in a new light. Genes Dev.

[9] Massoud TF, Gambhir SS (2007). Integrating noninvasive molecular imaging into molecular medicine: an evolving paradigm. Trends Mol Med.

[10] Frangioni JV (2008). New technologies for human cancer imaging. J Clin Oncol.

[11] Hu Z, Qu Y, Wang K, Zhang X, Zha J, Song T (2015). In vivo nanoparticle-mediated radiopharmaceutical-excited fluorescence molecular imaging. Nature Communications.

[12] Zhang Z, Cai M, Bao C, Hu Z, Tian J (2019). Endoscopic Cerenkov luminescence imaging and image-guided tumor resection on hepatocellular carcinoma-bearing mouse models. Nanomedicine: Nanotechnology, Biology and Medicine.

[13] Yang J, Zhao C, Lim J, Zhao L, Tourneau RL, Zhang Q (2021). Structurally symmetric near-infrared fluorophore IRDye78-protein complex enables multimodal cancer imaging. Theranostics.

[14] Yang J, Wang T, Zhao L, Rajasekhar VK, Joshi S, Andreou C (2020). Gold/alpha-lactalbumin nanoprobes for the imaging and treatment of breast cancer. Nat Biomed Eng.

[15] Te Velde EA, Veerman T, Subramaniam V, Ruers T (2010). The use of fluorescent dyes and probes in surgical oncology. European journal of surgical oncology: the journal of the European Society of Surgical Oncology and the British Association of Surgical Oncology.

[16] Kobayashi H, Ogawa M, Alford R, Choyke PL, Urano Y (2010). New Strategies for Fluorescent Probe Design in Medical Diagnostic Imaging. Chemical Reviews.

[17] Yi Z, Luo Z, Qin X, Chen Q, Liu X (2020). Lanthanide-Activated Nanoparticles: A Toolbox for Bioimaging, Therapeutics, and Neuromodulation. Accounts of Chemical Research.

[18] Lo ¨ıc J (2006). Charbonnie`re, † Niko Hildebrandt, Raymond F. Ziessel,and Hans-Gerd Lo ¨hmannsro ¨ben. Lanthanides to Quantum Dots Resonance Energy Transfer in Time-Resolved Fluoro-Immunoassays and Luminescence Microscopy. J AM CHEM SOC.

[19] Smith BR, Gambhir SS (2017). Nanomaterials for In Vivo Imaging. Chem Rev.

[20] Dong H, Du S-R, Zheng X-Y, Lyu G-M, Sun L-D, Li L-D (2015). Lanthanide Nanoparticles: From Design toward Bioimaging and Therapy. Chemical Reviews.

[21] Bünzli G (2010). Lanthanide Luminescence for Biomedical Analyses and Imaging. 2010.

[22] Tonga GY, Moyano DF, Kim CS, Rotello VM (2014). Inorganic Nanoparticles for Therapeutic Delivery: Trials, Tribulations and Promise. Curr Opin Colloid Interface Sci.

[23] D'Aléo A, Pointillart F, Ouahab L, Andraud C, Maury O (2012). Charge transfer excited states sensitization of lanthanide emitting from the visible to the near-infra-red. Coordination Chemistry Reviews.

[24] Liu Y, Zhou S, Tu D, Chen Z, Huang M, Zhu H (2012). Amine-functionalized lanthanide-doped zirconia nanoparticles: optical spectroscopy, time-resolved fluorescence resonance energy transfer biodetection, and targeted imaging. J Am Chem Soc.

[25] Fischer S, Siefe C, Swearer DF, McLellan CA, Alivisatos AP, Dionne JA (2020). Bright Infrared-to-Ultraviolet/Visible Upconversion in Small Alkaline Earth-Based Nanoparticles with Biocompatible CaF2 Shells. Angew Chem Int Ed Engl.

[26] Wang Z, Zhang T, Pi L, Xiang H, Dong P, Lu C (2021). Large-scale one-pot synthesis of water-soluble and biocompatible upconversion nanoparticles for dual-modal imaging. Colloids Surf B Biointerfaces.

[27] Plunkett S, El Khatib M, Sencan I, Porter JE, Kumar ATN, Collins JE (2020). In vivo deep-tissue microscopy with UCNP/Janus-dendrimers as imaging probes: resolution at depth and feasibility of ratiometric sensing. Nanoscale.

[28] Luo W, Zou DH, Yang S, Cui LN, Liu PY, Zhu QY (2019). Water-Soluble Lanthanide-Titanium-Oxo Cluster, a Precursor for Biocompatible Nanomaterial. Inorg Chem.

[29] Ren F, Liu H, Zhang H, Jiang Z, Xia B, Genevois C (2020). Engineering NIR-IIb fluorescence of Er-based lanthanide nanoparticles for through-skull targeted imaging and imaging-guided surgery of orthotopic glioma. Nano Today.

[30] Zhao M, Li B, Wu Y, He H, Zhu X, Zhang H (2020). A Tumor-Microenvironment-Responsive Lanthanide-Cyanine FRET Sensor for NIR-II Luminescence-Lifetime In Situ Imaging of Hepatocellular Carcinoma. Adv Mater.

[31] Xu J, Shi R, Chen G, Dong S, Yang P, Zhang Z (2020). All-in-One Theranostic Nanomedicine with Ultrabright Second Near-Infrared Emission for Tumor-Modulated Bioimaging and Chemodynamic/Photodynamic Therapy. ACS Nano.

[32] Zhang Y, Ma X, Chau H-F, Thor W, Jiang L, Zha S (2021). Lanthanide-Cyclen-Camptothecin Nanocomposites for Cancer Theranostics Guided by Near-Infrared and Magnetic Resonance Imaging. ACS Applied Nano Materials.

[33] Xu M, Yang G, Bi H, Xu J, Feng L, Yang D (2019). Combination of CuS and g-C3N4 QDs on upconversion nanoparticles for targeted photothermal and photodynamic cancer therapy. Chemical Engineering Journal.

[34] I YL, Jiang M, Xue Z, Zeng S (2020). 808 nm light triggered lanthanide nanoprobes with enhanced down-shifting emission beyond 1500 nm for imaging-guided resection surgery of tumor and vascular visualization. Theranostics.

[35] Feng M, Wang Y, Lin B, Peng X, Yuan Y, Tao X (2020). Degradable pH-responsive NIR-II imaging probes based on a polymer-lanthanide composite for chemotherapy. Dalton Trans.

[36] Cao J, Zhang L, Ding X, Liu D, Su B, Shi J (2020). Dual-Targeting Peptides RGD10-NGR9-Conjugated Lanthanide Nanoparticle@Polydopamine as Upconversion Nanoprobes for In Vivo Imaging of Lung Cancer. Small Methods.

[37] Li D, He S, Wu Y, Liu J, Liu Q, Chang B (2019). Excretable Lanthanide Nanoparticle for Biomedical Imaging and Surgical Navigation in the Second Near-Infrared Window. Adv Sci (Weinh).

[38] Shao Y, Liu B, Di Z, Zhang G, Sun LD, Li L (2020). Engineering of Upconverted Metal-Organic Frameworks for Near-Infrared Light-Triggered Combinational Photodynamic/Chemo-/Immunotherapy against Hypoxic Tumors. J Am Chem Soc.

[39] Zhong Y, Ma Z, Wang F, Wang X, Yang Y, Liu Y (2019). In vivo molecular imaging for immunotherapy using ultra-bright near-infrared-IIb rare-earth nanoparticles. Nat Biotechnol.

[40] Li Y, Zeng S, Hao J (2019). Non-Invasive Optical Guided Tumor Metastasis/Vessel Imaging by Using Lanthanide Nanoprobe with Enhanced Down-Shifting Emission beyond 1500 nm. ACS Nano.

[41] Zhang Q, Pratt EC, Tamura R, Ogirala A, Hsu HT, Farahmand N (2021). Ultrasmall Downconverting Nanoparticle for Enhanced Cerenkov Imaging. Nano Lett.

[42] Pratt EC, Shaffer TM, Zhang Q, Drain CM, Grimm J (2018). Nanoparticles as multimodal photon transducers of ionizing radiation. Nature Nanotechnology.

[43] Li Y, Lin J, Wang P, Luo Q, Lin H, Zhang Y (2019). Tumor Microenvironment Responsive Shape-Reversal Self-Targeting Virus-Inspired Nanodrug for Imaging-Guided Near-Infrared-II Photothermal Chemotherapy. ACS Nano.

[44] Xu L, Xue Y, Xia J, Qu X, Lei B, Yang T (2020). Construction of high quality ultrathin lanthanide oxyiodide nanosheets for enhanced CT imaging and anticancer drug delivery to efficient cancer theranostics. Biomaterials.

[45] Kuang Y, Zhang Y, Zhao Y, Cao Y, Zhang Y, Chong Y (2020). Dual-Stimuli-Responsive Multifunctional Gd2Hf2O7 Nanoparticles for MRI-Guided Combined Chemo-/Photothermal-/Radiotherapy of Resistant Tumors. ACS Appl Mater Interfaces.

[46] McLeod SM, Robison L, Parigi G, Olszewski A, Drout RJ, Gong X (2020). Maximizing Magnetic Resonance Contrast in Gd(III) Nanoconjugates: Investigation of Proton Relaxation in Zirconium Metal-Organic Frameworks. ACS Appl Mater Interfaces.

[47] Wang P, Hao L, Wang Z, Wang Y, Guo M, Zhang P (2020). Gadolinium-Doped BTO-Functionalized Nanocomposites with Enhanced MRI and X-ray Dual Imaging to Simulate the Electrical Properties of Bone. ACS Appl Mater Interfaces.

[48] Cai Y, Wang Y, Zhang T, Pan Y (2020). Gadolinium-Labeled Ferritin Nanoparticles as T1 Contrast Agents for Magnetic Resonance Imaging of Tumors. ACS Applied Nano Materials.

[49] Babu S, Cho JH, Dowding JM, Heckert E, Komanski C, Das S (2010). Multicolored redox active upconverter cerium oxide nanoparticle for bio-imaging and therapeutics. Chem Commun (Camb).

[50] D'Achille AE, Gonzalez-Rodriguez R, Campbell E, Lee BH, Coffer JL, Naumov AV (2020). Rare-Earth-Doped Cerium Oxide Nanocubes for Biomedical Near-Infrared and Magnetic Resonance Imaging. ACS Biomater Sci Eng.

[51] Shao C, Shen A, Zhang M, Meng X, Song C, Liu Y (2018). Oxygen Vacancies Enhanced CeO2:Gd Nanoparticles for Sensing a Tumor Vascular Microenvironment by Magnetic Resonance Imaging. ACS Nano.

[52] Wei Z, Wu M, Li Z, Lin Z, Zeng J, Sun H (2018). Gadolinium-doped hollow CeO2-ZrO2 nanoplatform as multifunctional MRI/CT dual-modal imaging agent and drug delivery vehicle. Drug Deliv.

[53] Banerjee S, Pillai MR, Knapp FF (2015). Lutetium-177 therapeutic radiopharmaceuticals: linking chemistry, radiochemistry, and practical applications. Chem Rev.

[54] Viana RdS, Costa LAdM, Harmon AC, Gomes Filho MA, Falcão EHL, Vicente MGH (2020). 177Lu-Labeled Eu-Doped Mesoporous SiO2 Nanoparticles as a Theranostic Radiopharmaceutical for Colorectal Cancer. ACS Applied Nano Materials.

[55] Yu B, Wei H, He Q, Ferreira CA, Kutyreff CJ, Ni D (2018). Efficient Uptake of (177) Lu-Porphyrin-PEG Nanocomplexes by Tumor Mitochondria for Multimodal-Imaging-Guided Combination Therapy. Angew Chem Int Ed Engl.

[56] Pei P, Shen W, Zhou H, Sun Y, Zhong J, Liu T (2021). Radionuclide labeled gold nanoclusters boost effective anti-tumor immunity for augmented radio-immunotherapy of cancer. Nano Today.

[57] Chen Z, Zhang Z, Zhai X, Li Y, Lin L, Zhao H (2020). Rapid and Sensitive Detection of anti-SARS-CoV-2 IgG, Using Lanthanide-Doped Nanoparticles-Based Lateral Flow Immunoassay. Anal Chem.

[58] Zhao M, Wang R, Li B, Fan Y, Wu Y, Zhu X (2019). Precise In Vivo Inflammation Imaging Using In Situ Responsive Cross-linking of Glutathione-Modified Ultra-Small NIR-II Lanthanide Nanoparticles. Angew Chem Int Ed Engl.

[59] Pinol R, Zeler J, Brites CDS, Gu Y, Tellez P, Carneiro Neto AN (2020). Real-Time Intracellular Temperature Imaging Using Lanthanide-Bearing Polymeric Micelles. Nano Lett.

[60] Pan Y, Xie X, Huang Q, Gao C, Wang Y, Wang L (2018). Inherently Eu(2+) /Eu(3+) Codoped Sc2 O3 Nanoparticles as High-Performance Nanothermometers. Adv Mater.

[61] Liu J, Pan L, Shang C, Lu B, Wu R, Feng Y (2020). A highly sensitive and selective nanosensor for near-infrared potassium imaging. Science Advances.

[62] Galaup C, Picard C, Couderc F, Gilard V, Collin F (2021). Luminescent lanthanide complexes for reactive oxygen species biosensing and possible application in Alzheimer's diseases. FEBS J.

[63] Charpentier C, Cifliku V, Goetz J, Nonat A, Cheignon C, Cardoso Dos Santos M (2020). Ultrabright Terbium Nanoparticles for FRET Biosensing and in Situ Imaging of Epidermal Growth Factor Receptors*. Chemistry.

[64] He S, Chen S, Li D, Wu Y, Zhang X, Liu J (2019). High Affinity to Skeleton Rare Earth Doped Nanoparticles for Near-Infrared II Imaging. Nano Lett.

[65] Neufeld MJ, Winter H, Landry MR, Goforth AM, Khan S, Pratx G (2020). Lanthanide Metal-Organic Frameworks for Multispectral Radioluminescent Imaging. ACS Appl Mater Interfaces.

[66] Cardoso Dos Santos M, Runser A, Bartenlian H, Nonat AM, Charbonnière LJ, Klymchenko AS (2019). Lanthanide-Complex-Loaded Polymer Nanoparticles for Background-Free Single-Particle and Live-Cell Imaging. Chemistry of Materials.

[67] Jia Q, Ma L, Zhai X, Fu W, Liu Y, Liao X (2020). Orthogonal Near-Infrared-II Imaging Enables Spatially Distinguishing Tissues Based on Lanthanide-Doped Nanoprobes. Anal Chem.

[68] Huang J, Li J, Zhang X, Zhang W, Yu Z, Ling B (2020). Artificial Atomic Vacancies Tailor Near-Infrared II Excited Multiplexing Upconversion in Core-Shell Lanthanide Nanoparticles. Nano Lett.

[69] Deng Z, Huang J, Xue Z, Jiang M, Li Y, Zeng S (2020). A general strategy for designing NIR-II emissive silk for the in vivo monitoring of an implanted stent model beyond 1500 nm. J Mater Chem B.

[70] Hofmann-Amtenbrink M, Grainger DW, Hofmann H (2015). Nanoparticles in medicine: Current challenges facing inorganic nanoparticle toxicity assessments and standardizations. Nanomedicine: Nanotechnology, Biology and Medicine.

[71] Rogosnitzky M, Branch S (2016). Gadolinium-based contrast agent toxicity: a review of known and proposed mechanisms. Biometals.

[72] Gnach A, Lipinski T, Bednarkiewicz A, Rybka J, Capobianco JA (2015). Upconverting nanoparticles: assessing the toxicity. Chem Soc Rev.

[73] Hirano S, Suzuki KT (1996). Exposure, metabolism, and toxicity of rare earths and related compounds. Environ Health Perspect.

[74] Samhadaneh DM, Mandl GA, Han Z, Mahjoob M, Weber SC, Tuznik M (2020). Evaluation of Lanthanide-Doped Upconverting Nanoparticles for in Vitro and in Vivo Applications. ACS Applied Bio Materials.

[75] Xing H, Bu W, Ren Q, Zheng X, Li M, Zhang S (2012). A NaYbF4: Tm3+ nanoprobe for CT and NIR-to-NIR fluorescent bimodal imaging. Biomaterials.

[76] Yan J, He W, Yan S, Niu F, Liu T, Ma B (2018). Self-Assembled Peptide-Lanthanide Nanoclusters for Safe Tumor Therapy: Overcoming and Utilizing Biological Barriers to Peptide Drug Delivery. ACS Nano.

